# Practical use and underlying physics of the BENCHMARK™ BMX™ 96 for large-bore aspiration thrombectomy: Case report of initial institutional experience

**DOI:** 10.1177/19714009211036691

**Published:** 2021-08-03

**Authors:** Ian R Macdonald, Elena A Cora, Ian Grant, David Volders

**Affiliations:** 1 Division of Neuroradiology, Department of Diagnostic Radiology, 3688 Dalhousie University, Canada; 2 Division of Neurology, Department of Medicine, Dalhousie University, Canada

**Keywords:** Interventional neuroradiology, endovascular thrombectomy, BENCHMARK™ BMX™ 96, A Direct Aspiration first Pass Technique (ADAPT)

## Abstract

Endovascular thrombectomy (EVT) is part of first-line intervention for acute ischemic stroke management. Recent technological advances have demonstrated that large-bore catheters are an attractive approach for EVT. A multitude of approaches such as A Direct Aspiration first Pass Technique (ADAPT) or in conjunction with stent retrieval (Solumbra technique) have been developed with increasingly large-bore catheters, demonstrating safety and efficacy. Furthermore, these techniques have demonstrated promise for the intervention of cerebral venous thrombosis as well as posterior circulation ischemic events. Recently, advances in neurointerventional catheters have focused on improved maneuverability to navigate the neurovasculature, as well as larger inner diameters for improved procedural versatility, including aspiration. We describe a case report highlighting our early institutional experience with the recently developed large-bore catheter, the BENCHMARK™ BMX™ 96. The case report entails near complete occlusion of the internal carotid artery from acute thrombus and the utility of the BMX™ 96 catheter for treatment of such extensive clot burden. The applicability of large-bore aspiration catheters, with an emphasis on recent advances, for mechanical thrombectomy in arterial as well as venous systems is discussed. To our knowledge, this is the first reported case of use of the BENCHMARK™ BMX™ 96 access system for EVT in acute ischemic stroke. Such new-generation large-bore catheters are a promising advance in neurointervention, and our early institution experience highlights the ease of use and versatility for neurointerventional procedures such as EVT.

## Introduction

Endovascular thrombectomy (EVT) is a mainstay of acute ischemic stroke management and is considered part of first-line intervention as supported by recent guidelines,^1^ with a diverse range of techniques described.^2^ Continued recent technological advances have positioned increasingly large-bore catheters as an attractive approach for EVT in ischemic stroke. These catheters are typically used either in the setting of A Direct Aspiration first Pass Technique (ADAPT)^3^ or in conjunction with stent retrieval (Solumbra technique).^4^ Stand-alone large-bore catheter aspiration has also shown efficacy for cerebral venous thrombosis^5^ as well as posterior circulation EVT.^6^ Recent advances in neurointerventional catheters have been focused on improved system maneuverability and conformability to optimize navigation of the neurovasculature. Furthermore, larger inner diameters (ID) improve procedural versatility, such as aspiration, which has translated into safe and efficacious ADAPT approaches for acute ischemic stroke.^7,8^ Recent clinical trials (COMPASS^9^ and ASTER^10^) have positioned ADAPT as an attractive first-line approach for EVT. We describe a case report highlighting our early institutional experience with the recently developed large-bore aspiration catheter, the BENCHMARK™ BMX™ 96 (Penumbra, Inc., Alameda, CA).

## Case

**Figure 1.**
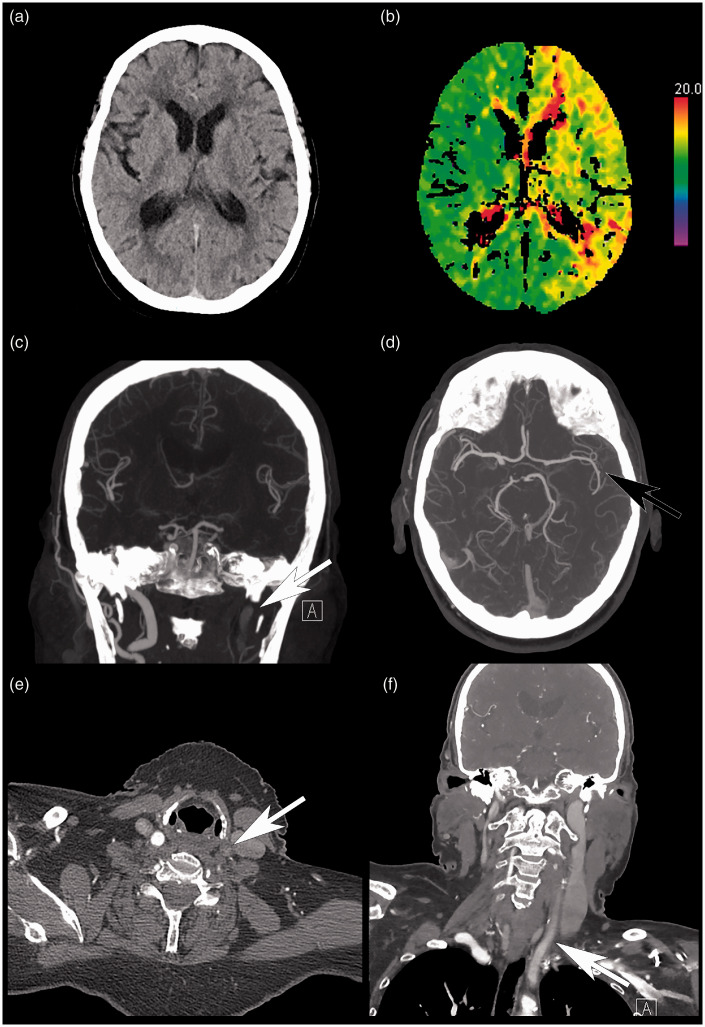
Computed tomography (CT) scan of the head, time-to-peak perfusion map, and CT angiogram study. (a) Select unenhanced axial CT head slice, demonstrating no evidence of infarct (ASPECTS 10). (b) Corresponding time-to-peak perfusion map, demonstrating diffuse abnormality in the left middle cerebral artery territory. (c) Coronal maximum intensity projection (MIP), demonstrating occlusion of the left internal carotid artery (ICA; white arrow). (d) Axial MIP, demonstrating persistent filling of the left middle cerebral artery (black arrow) secondary to collateral flow. Select axial (e) and coronal (f) CT angiogram slices, demonstrating slow flow at the level of the mid-left common carotid artery (CCA; white arrows). Note that the patient clinically demonstrated right hemiparesis as well as aphasia.

A 66-year-old female presented to an outside hospital three hours after acute stroke symptom onset with right hemiplegia and aphasia, with a National Institutes of Health Stroke Scale (NIHSS) of 12. A non-contrast computed tomography (NCCT) scan of the head confirmed preserved gray–white differentiation, with no signs of early infarction or hemorrhage (Alberta Stroke Program Early CT Score (ASPECTS) 10). A CT angiogram (CTA) of the arch to the vertex revealed absent contrast filling from the mid-portion of the left common carotid artery (CCA) to the supraclinoid segment of the left internal carotid artery (ICA). The left middle cerebral artery (MCA) circulation was preserved secondary to collateral flow via the anterior communicating artery. Intravenous thrombolytic therapy was contraindicated, as the patient was taking warfarin for atrial fibrillation (International Normalized Ratio 2.3). Given the patient’s excellent ASPECTS score, good collaterals, and moderate stroke symptoms (a NIHSS score of 12), the patient was transported to our comprehensive stroke center for emergency EVT consideration. After arrival, approximately four hours later, a repeat NCCT scan of the head and CTA of the arch to the vertex showed no interval change, with no signs of early infarction (ASPECTS 10) and persistent left ICA occlusion (Figure 1). Additional CT perfusion (CTP) of the brain showed decreased perfusion to the left MCA territory (Figure 1). Given the persistent moderate stroke symptoms (a NIHSS score of 12) and the significant penumbra on imaging, the patient was urgently transferred to the angiography suite for an emergency EVT.

**Figure 2.**
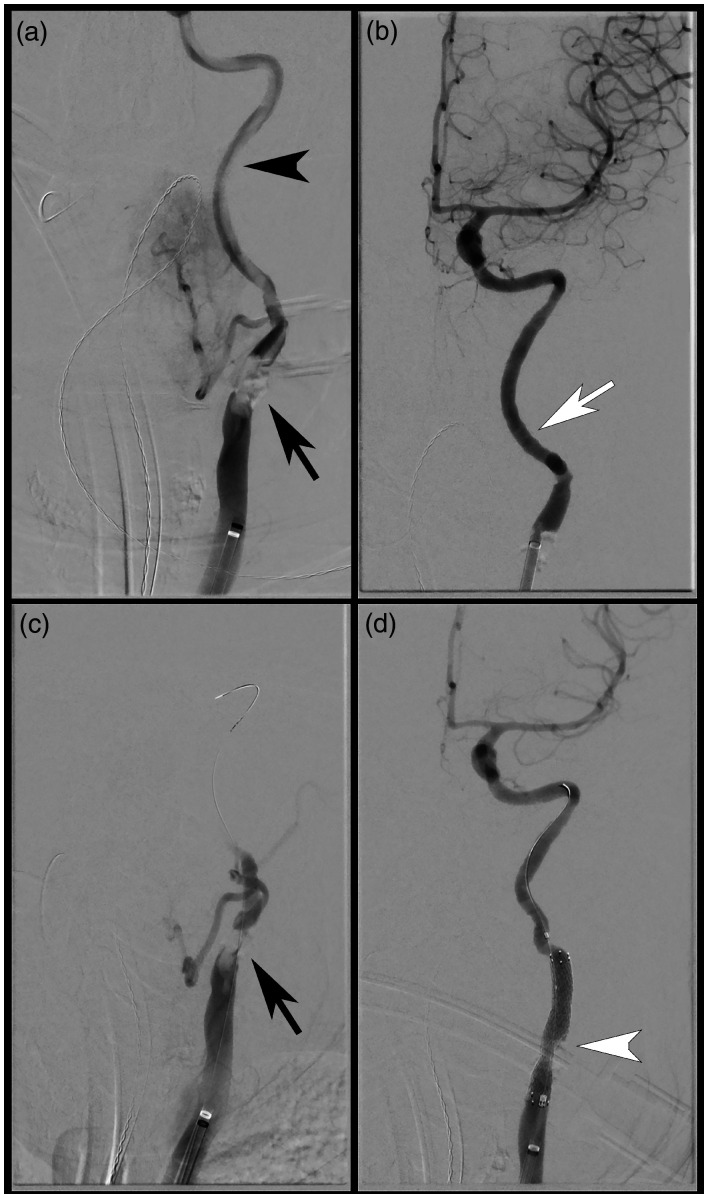
Endovascular thrombectomy (EVT) and stent deployment. (a) Anterior-posterior digital subtraction angiogram with BMX™ 96 catheter positioned in the mid-left CCA, demonstrating severe stenosis at the left CCA bifurcation (black arrow), with extensive near occlusive thrombus throughout the left ICA (black arrowhead). (b) Post aspiration thrombectomy. demonstrating widely patent left ICA with no distal intracranial emboli (TICI 3; white arrow). (c) Persistent severe left carotid bifurcation stenosis post EVT (black arrow). (d) Angiogram post stent and angioplasty, demonstrating patency of the left carotid bifurcation (white arrowhead).

**Figure 3.**
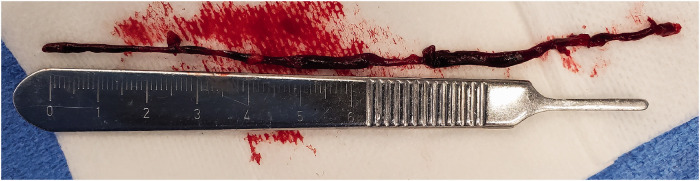
Thrombectomy specimen. Photo of the aspirated left common carotid/ICA thrombus measuring 13 cm in length, obtained after one pass of the BECNHMARK™ BMX™ 96 aspiration catheter.

General anesthesia was induced, and the patient was prepped in standard fashion. After right common femoral artery access, a co-axial system of a 90 cm BENCHMARK™ BMX™ 96 access catheter and 125 cm 6F Bern select catheter (Penumbra, Inc.) was easily maneuvered into the mid left CCA over a Terumo Advantage guidewire (Terumo Medical Canada, Inc., Vaughan, Canada). Angiographic runs demonstrated near occlusion of the carotid bifurcation with a string sign and large amount of thrombus throughout the left ICA (Figure 2(a)). The left ICA was successfully selected, and the BMX™ 96 catheter was positioned in the mid-cervical ICA, with no issues navigating the severe bifurcation stenosis. No spontaneous back flow was noted from the BMX™ 96 after removal of the hemostatic valve. Aspiration tubing paired with a ENGINE aspiration pump (Penumbra, Inc.) was connected to the BMX™ 96, and aspiration was applied for two minutes. Subsequently, 13 cm of thrombus was spontaneously aspirated through the BMX™ 96 and its connecting tube to the pump without manipulating the catheter (Figure 3). A subsequent angiogram showed complete recanalization of the left ICA, with no evidence of distal emboli (TICI 3; Figure 2(b)). The time from groin puncture to first recanalization was 15 minutes. The BMX™ 96 was subsequently pulled back to the proximal cervical ICA (Figure 2(c)), and aspiration at this level yielded additional small pieces of thrombus. The patient was loaded with 325 mg aspirin and 300 mg clopidogrel through an OG tube. Carotid bifurcation stenting using a tapered 8–6 mm × 40 mm EV3 Protégé RX stent (Medtronic, Minneapolis, MN) and balloon angioplasty using an Armada 18 4 mm × 20 mm balloon (Abbott, Plymouth, MN) was successfully performed. A control left CCA angiogram showed satisfactory result with no residual stenosis (Figure 2(d)). Hemostasis of the right groin was achieved through manual compression for 30 minutes. The patient was extubated and transported to our recovery room, with subsequent transfer to home hospital several hours later. Twenty-four hours post procedure, the patient had neurologically returned to normal, with no residual weakness or aphasia (a NIHSS score of 0).

## Discussion

**Figure 4.**
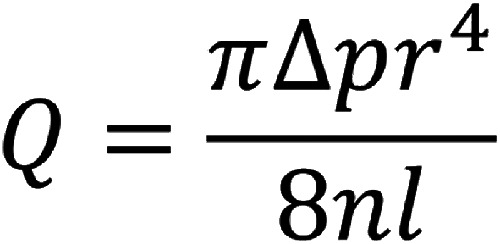
Poiseuille’s law rearrangement, highlighting the relationship between volumetric flow rate (*Q*) and radius (*r*) to the 4th power. Small increases in aspiration catheter inner diameter can thus translate into significant increases in force for endovascular thrombectomy. *n* = fluid viscosity, *l* = length of tubing, Δ*p* = pressure difference.

This case report highlights the use of a BMX™ 96 catheter in the setting of extensive arterial clot burden. The BMX™ 96 has hydrophilic coating along the distal 11 cm, as well as progressive laser-cut patterns along the entire length, contributing to the distal ease of maneuverability at the level of the aortic arch and cervical ICA while balancing proximal support. This can reduce the possible need to perform an exchange maneuver and therefore translates into reduced procedure times. The BMX™ 96 has an 8 Fr outer diameter (OD; 0.109 inch) and a 0.096 inch ID. To our knowledge, this ID is the largest currently available for aspiration catheters used in EVT treatment and has favorable dimensions compared to other widely used aspiration catheters such as Neuron Max (Penumbra, Inc.; OD of 0.112 inch and ID of 0.088 inch). The larger ID in the setting of ADAPT conceptually provides capacity to aspirate a greater thrombus burden from larger vessels such as the ICA with minimal repositioning and potentially minimal passes, as recently supported in a meta-analysis.^11^ When paired with a suitable vacuum pump, even small increases in catheter ID translate into exponentially greater force applied to the thrombus for removal. Considering Poiseuille’s law, the rate of flow through a pipe for steady flow relates to the pressure difference across the two ends of the pipe, the radius of the tube, as well as the fluid viscosity and the length of the tube. In particular, the radius has a direct relationship to the 4th power to the flow (Figure 4). When confronted with a large ICA clot burden, the BMX™ 96 can provide an opportunity to aspirate large amounts of thrombus, without the need to pass first with a microcatheter/microwire combination, thereby likely reducing the risk of dislodging distal emboli. Furthermore, techniques using the combination of EVT with a proximal balloon guide catheter (BCG) have emerged^1^ to minimize the risk of potential distal emboli. However, much of this evidence predates recent large-bore aspiration catheters. More recent BCG studies using Sofia catheter systems (MicroVention, Aliso Viejo, C; up to 0.070 inch ID) demonstrate efficacy and shorter procedure times in conjunction with large-bore aspiration catheters.^12,13^ Additionally, the aspiration catheter size positively correlates with the rate of first-pass effect when used in conjunction with BCG and stent retrieval.^14^ However, the IDs of some of the larger BCG systems such as the MERCI 9F (Stryker, Kalamazoo, MI; ID of 0.085 inch) remain smaller than the BMX™ 96 system, raising the issue of compatibility with large-bore catheters. With increasing advances in large-bore aspiration catheters, the need for larger-bore BCGs may be needed.

Large-bore aspiration catheters are also a promising approach for the treatment of cerebral venous thrombosis, which otherwise has high morbidity and mortality rates, with a proclivity for younger patients. Small case series demonstrate the utility and safety of large-bore aspiration catheters in cerebral venous thrombosis.^15^ In particular, given the association of cerebral sinus thrombosis with infections, such as coronavirus disease 2019, continued refinement of approaches for effective treatment is urgently required. One of the additional benefits of a large-bore aspiration system, such as the BMX™ 96, in the setting of ADAPT is the reduced cost compared to more traditional catheters that are typically used in conjunction with stents and/or BCGs. Although promising, further analysis is warranted to analyze the efficacy and complication risk of larger-bore neurointerventional catheters as they gain more widespread use.

Finally, management approaches for symptomatic acute carotid artery occlusions with preserved circle of Willis flow, as demonstrated in our case report, are not well established. The natural history of such occlusions may be more favorable due to collateral flow via the circle of Willis, thus arguing against placing the patient at potential risk of procedure-related complications. However, these patients remain at increased risk of infarct, particularly in watershed regions. A recent retrospective analysis, using the MR CLEAN registry, suggests generally improved clinical outcomes for patients who underwent EVT for isolated intracranial ICA occlusions. However, 20% of these patients did demonstrate distal clot migration with subsequent poor clinical outcome.^16^ The use of an ADAPT approach with a large-bore catheter may minimize the risk of intervention in these patients. Future studies, ideally using the most recent large-bore catheters, will likely aid to elucidate further safe and efficacious management options for patients with isolated acute carotid occlusions.

## Conclusion

The BENCHMARK™ BMX™ 96 access system is a promising advance in neurointervention, and to our knowledge, this is the first reported case of use in ischemic stroke. Our early institution experience highlights the ease of use and versatility for neurointerventional procedures such as EVT.
